# Diagnostic Approach and Pathological Characterization of Metastatic Intrahepatic Cholangiocarcinoma in a Captive Puma (*Puma concolor*)

**DOI:** 10.3390/ani15121821

**Published:** 2025-06-19

**Authors:** Elisa Mazzotta, Claudia Zanardello, Giovanni De Zottis, Antonio Barberio, Mery Campalto, Federico Martignago, Giulia Maria De Benedictis, Carlo Guglielmini, Francesca Zanusso, Greta Foiani

**Affiliations:** 1 Istituto Zooprofilattico Sperimentale delle Venezie (IZSVe), Viale dell’Università 10, 35020 Legnaro, PD, Italy; abarberio@izsvenezie.it (A.B.); mcampalto@izsvenezie.it (M.C.); fmartignago@izsvenezie.it (F.M.); 2 Laboratory of Histopathology (SCS3), Istituto Zooprofilattico Sperimentale delle Venezie (IZSVe), Viale dell’Università 10, 35020 Legnaro, PD, Italy; czanardello@izsvenezie.it (C.Z.); gdzottis@izsvenezie.it (G.D.Z.); 3 Department of Animal Medicine, Productions and Health (MAPS), University of Padua, Viale dell’Università 12, 35020 Legnaro, PD, Italy; giuliamaria.debenedictis@unipd.it (G.M.D.B.); carlo.guglielmini@unipd.it (C.G.); francesca.zanusso@studenti.unipd.it (F.Z.)

**Keywords:** captive felids, cholangiocarcinoma, clinical investigation, immunohistochemistry, metastatic, *Puma concolor*

## Abstract

A 16-year-old captive male puma (*Puma concolor*) presented with progressive weight loss and acute mild gastrointestinal symptoms. Clinical examination, and diagnostic imaging revealed abdominal, pleural, and pericardial effusion and nodular masses in multiple organs. Due to the clinical condition, multiple organ involvement, and a poor prognosis, compassionate euthanasia was chosen. Necropsy revealed abundant cavitary effusions and multiple yellow–white neoplastic masses involving the left hepatic lobes. Metastatic nodules were observed throughout the peritoneum, pericardium, kidney, spleen, and spermatic cord. Histologically, the tumor was characterized by tubular and acinar structures embedded in a desmoplastic stroma. Neoplastic cells were positive for pan-cytokeratins (CKs), CK7, and, to a lesser extent, CK20. The gross, histologic, and immunohistochemical findings were consistent with intrahepatic metastatic cholangiocarcinoma.

## Simple Summary

This report describes a case of intrahepatic cholangiocarcinoma with disseminated metastasis in a 16-year-old captive puma, highlighting the value of a comprehensive, multidisciplinary approach. It underscores the importance of clinical, diagnostic, and pathological collaboration in fully understanding the disease. The case provides important insights for veterinarians, pathologists, and wildlife specialists, emphasizing the need for integrated efforts in diagnosing and managing complex conditions in large felids under human care.

## 1. Introduction

Cholangiocarcinomas (CCAs) are primary tumors of the biliary tract, originating from the intrahepatic or extrahepatic bile ducts or the gallbladder. These tumors have been reported in both domestic and wild animals [1,2], including captive large felids such as lions (*Panthera leo*) [3,4,5], leopards (*Panthera pardus*) [4,5], and tigers (*Panthera tigris*) [4], with only a single case documented in pumas (*Puma concolor*) [6]. In these species, CCAs are described as highly aggressive neoplasms, frequently metastasizing to distant organs. Most reports in wild felids consist of case series or surveys from a zoologic institution, primarily focusing on post-mortem pathological findings [4,5]. In this report, we aim to document the diagnostic approach that led to the identification of a metastatic intrahepatic CCA in an elderly captive puma, including clinical evaluation, diagnostic imaging, and collateral microbiologic analysis, and to provide a characterization of its pathological features, highlighting the importance of a multidisciplinary approach to the management of these animals under human care.

## 2. Case Presentation

### 2.1. Clinical Report

A 16-year-old captive puma (*Puma concolor*, Linnaeus, 1771) presented with progressive weight loss, two days of decreased appetite, acute mild gastrointestinal signs (vomiting and diarrhea), listlessness, and reduced interaction. The animal came from a private wildlife collection comprising approximately 25 large felids. The puma used to live with two female pumas in an enclosure equipped with a nighttime shelter and various environmental enrichments (trees, a water pool, ropes, benches, stairs, etc.). The animal was routinely fed thermally treated raw meat, including rabbit, chicken, duck, and bovine sources. There were no relevant findings in the clinical history. Four years previously, a coprological examination had been positive for *Toxascaris leonina*, and the animal had received antiparasitic treatment accordingly and was annually checked for gastrointestinal parasites, with all subsequent controls testing negative. The animal was not regularly vaccinated. On visual examination, the animal was alert, ambulatory, and responsive to external stimuli. Abdominal distension was observed. Tachypnea and shallow respiration were noted with a respiratory rate of 32 breaths per minute. In order to perform a complete clinical evaluation, the animal was transferred to the Veterinary Teaching Hospital of the University of Padua (OVUD), where it was remotely anesthetized via intramuscular administration of a combination of dexmedetomidine (Dexdomitor, Vétoquinol Italia S.r.l., Forlì, Italy, 3 µg/kg), ketamine (Nimatek, Dechra Veterinary Products S.r.l., Torino, Italy, 2.5 mg/kg), midazolam (Midazolam Ibi, Ibi Istituto Biochimico Italiano Giovanni Lorenzini S.p.A., Aprilia, Italy, 0.2 mg/kg), and butorphanol (Dolorex, MSD Animal Health S.r.l., Segrate, Italy, 0.2 mg/kg). General anesthesia was maintained with an intravenous infusion of propofol (Proposure, Zoetis, Rome, Italy) to ensure an adequate depth of anesthesia throughout the diagnostic procedure. The animal was intubated, and supplemental oxygen was provided via an endotracheal tube. Physiological parameters were continuously monitored, and intravenous fluid therapy with Lactated Ringer’s solution (Ringer Lattato, Salf S.p.A., Cenate Sotto, Italy) was administered at a rate of 2 mL/kg/h.

**Figure 1:**
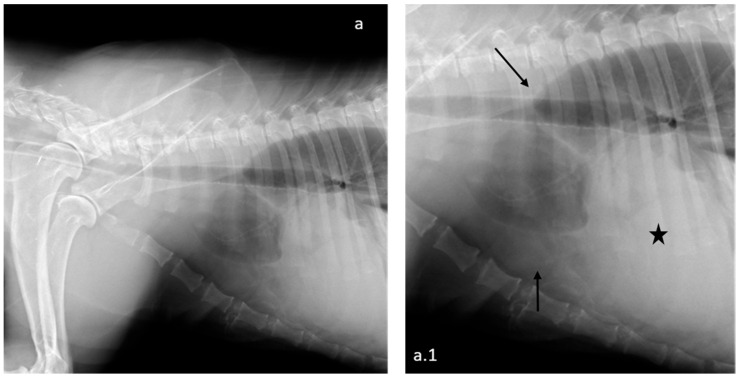
Thoracic radiographies: right lateral view (**a**) and details (**a.1**). The radiographic study is suggestive of severe bilateral pleural effusion, with dorsal mediastinal displacement and reduced pulmonary volume (arrows). Cardiac silhouette enlargement and increased radiopacity in the pericardial region are suggestive of pericardial effusion (star).

On admission, the animal weighed 55.4 kg, BCS < 3 [7,8], had marked abdominal distension, and was mildly dehydrated (estimated at approximately 5–6% of body weight loss based on clinical signs including delayed skin tenting and sticky mucous membranes). The mucous membranes were pale with a slightly increased capillary refill time (5 s). The popliteal lymph nodes were mildly enlarged, and both testicles appeared nodular and irregular on palpation. A complete blood count and serum biochemistry profile were performed, and the results reported a mild increase in the red blood cells count and hematocrit values (Table 1). Thoracic radiographies showed bilateral pleural and pericardial effusions (Figure 1).

An APOCUS (Abdominal Point-Of-Care Ultrasound Scan) and an echocardiography were also performed. The APOCUS showed the presence of a large amount of free fluid in the abdominal cavity. The ultrasound examination also showed fluid accumulation in the thoracic cavity and pericardial space. Abdominal ultrasonography revealed hypoechoic masses within the left hepatic lobe, with heterogeneous internal echotexture and irregular margins, suggestive of a neoplastic process. Nodular lesions in the spleen and right testicle were detected.

**Figure 2:**
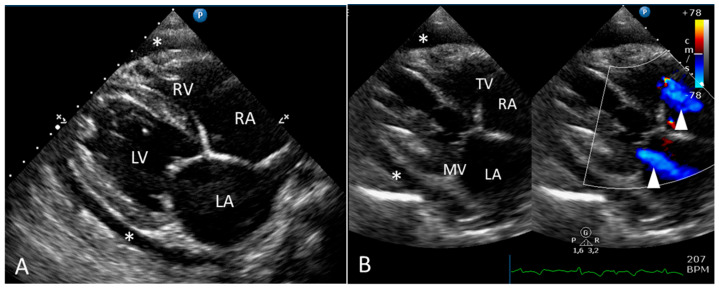
Two dimensional (2D) echocardiographic image (**A**) and combined 2D and color Doppler image (**B**) obtained from the right parasternal long axis view. The maximum depths in (**A**,**B**) are 16 cm and 15 cm, respectively. Moderate pericardial effusion (asterisks) and bi-atrial enlargement are evident (**A**). On Doppler interrogation of mitral and tricuspid blood flow (**B**), mild–moderate systolic regurgitant flow is visible (arrowheads). LA: left atrium; LV: left ventricle; MV: mitral valve; RA: right atrium; RV: right ventricle; TV: tricuspid valve.

On the Two-dimensional echocardiography, moderate pericardial effusion and moderate bi-atrial enlargement were visible (Figure 2). Other findings included pleural effusion associated with a distended caudal vena cava. A Doppler evaluation of transvalvular blood flow revealed mild–moderate mitral and tricuspid regurgitation. The combined absence of a recognizable P wave and irregular QRS complexes on simultaneous ECG tracing and the absence of the diastolic A wave on the pulsed-wave Doppler examination of the trans-mitral blood flow was suggestive of atrial fibrillation. No intrapericardial masses were visible.

Given the animal’s serious clinical condition and the lack of viable therapeutic options to control or manage disease progression, euthanasia was chosen on compassionate grounds. The animal, anesthetized using the aforementioned protocol, was brought to a deep level of anesthesia and was subsequently administered a licensed euthanasia agent (Embutramide + Mebezonium Iodide + Tetracaine (Tanax^®^, MSD Animal Health S.r.l., Segrate, Italy) in accordance with animal welfare guidelines. Subsequently, the carcass was sent to the laboratories of the Istituto Zooprofilattico Sperimentale delle Venezie for necropsy and post-mortem diagnostic investigations.

### 2.2. Gross Pathology

**Figure 3:**
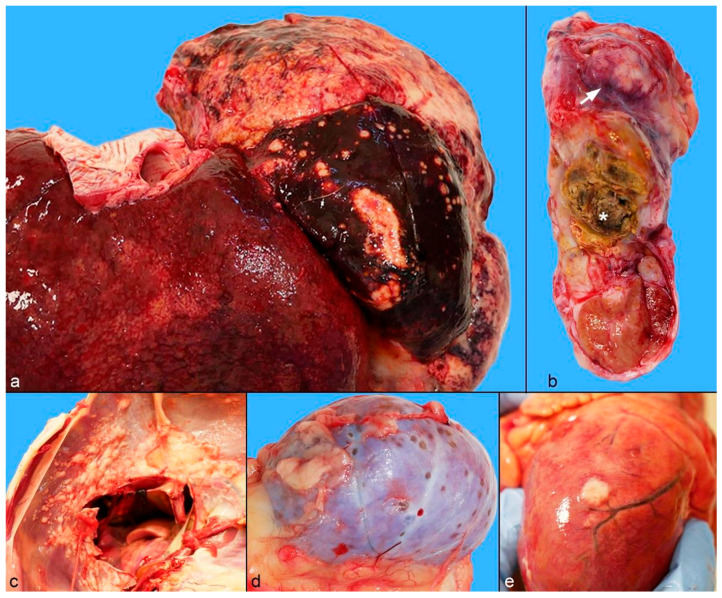
Gross findings of intrahepatic cholangiocarcinoma with disseminated metastases in a captive puma. (**a**) Whitish-yellow neoplastic masses in the left hepatic lobes. The left lateral lobe is severely enlarged and almost completely replaced by neoplasia. Multiple coalescing nodules, the largest with central depression and an umbilicated appearance, are visible in the left middle lobe. (**b**) Multiple whitish neoplastic nodules (arrow) are present within the spermatic cord, which is irregularly thickened, with an area of steatonecrosis (asterisk) and testis displacement. (**c**) Multiple coalescing whitish neoplastic nodules on the abdominal surface of the diaphragm. (**d**) Multiple small, depressed nodules on the cortical surface of the kidney. (**e**) Neoplastic nodule on the epicardium.

At necropsy, approximately 2 L of abdominal sero-hemorrhagic effusion were observed. The left lateral and middle hepatic lobes were affected by whitish-yellow firm neoplastic masses (Figure 3a). The lateral lobe was severely enlarged and almost entirely occupied by the neoplasm (massive appearance), measuring 17.5 × 12 × 4 cm. The middle lobe was characterized by multiple coalescing nodules, with the largest (3.5 × 2 × 1.5 cm) displaying an umbilicated appearance. Small nodules (up to 1.5 cm in diameter) were also noted on the abdominal and pleural surfaces of the diaphragm (Figure 3c), on the mesentery, and on the surface of the spleen. The right spermatic cord was severely thickened due to the whitish nodular masses (Figure 3b), causing a displacement of the right testis and a focal irregular area characterized by a yellowish—brown and dry appearance. Mild catarrhal gastritis with hemorrhage, and catarrhal enteritis were noted. The kidneys showed multiple small, depressed nodules (2–3 mm) on the cortical surface (Figure 3d). Abundant sero-hemorrhagic pleural and moderate pericardial effusions were present in the thoracic cavity. Multiple neoplastic nodules were detected on both the visceral (Figure 3e) and parietal pericardial surfaces, with partial infiltration into the wall of the cranial vena cava. No gross lesions were observed on the central nervous system.

### 2.3. Cytological, Histological, and Immunohistochemical Findings

At necropsy, smears from the pericardial effusion were prepared for cytological analysis and stained with May Grünwald-Giemsa quick stain (MGG Quick Stain, code 04-090805M, Bio-Optica Milano S.p.A., Milan, Italy). Tissue samples from the liver, diaphragm, spermatic cord, testis, kidney, spleen, and heart were fixed in 10% neutral buffered formalin and processed for routine histopathologic examination.

Automated immunohistochemistry (IHC) was performed on the Discovery ULTRA system (Roche, Ventana Medical Systems Inc., Tucson, AZ, USA) using the primary antibodies anti-pan cytokeratin (CK, clone AE1-AE3, code M3515, Dako, Glostrup, Denmark), CK7 (clone OV-TL, code M7018, Dako), CK20 (clone Ks20.8, code M7019, Dako), and the von Willebrand factor (vWF, polyclonal antibody, code A0082, Dako, Glostrup, Denmark). Detailed information about the IHC protocols is listed in Table 2. After detection, sections were counterstained with Mayer’s hematoxylin (Hematoxylin II, Ventana, Roche Diagnostics S.p.A., Monza, Italy).

**Figure 4:**
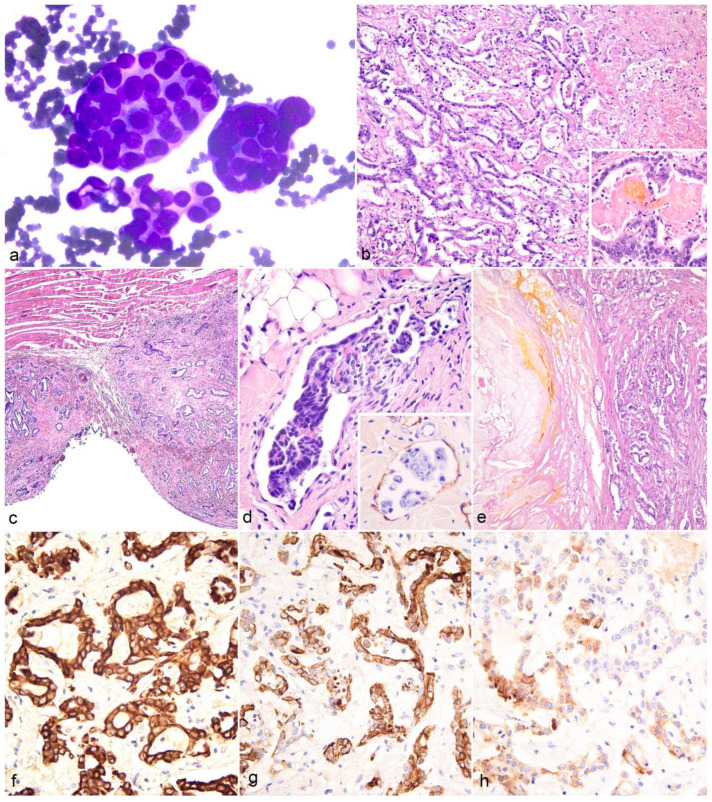
Cytologic, histologic, and immunohistochemical findings of intrahepatic cholangiocarcinoma with disseminated metastases in a captive puma. (**a**) Clusters of epithelial neoplastic cells in cytological smears from the pericardial effusion, found using May Grünwald-Giemsa staining. (**b**) Liver: irregular neoplastic tubular structures lined a cuboidal to low columnar epithelium, with lytic necrosis and hemorrhage (right) and occasional intraluminal yellowish material, consistent with biliary pigment (inset). Hematoxylin and Eosin (H&E) stains: (**c**) neoplastic metastatic nodules on the diaphragmatic surface consisting of tubular and acinar structures and desmoplastic stroma. (**d**) Lymphovascular invasion of neoplastic cells within the vessels of the diaphragm (H&E), with endothelial immunohistochemical staining with the von Willebrand factor (inset, DAB Chromogen). (**e**) Neoplastic infiltration of the spermatic cord, adjacent to an area of steatonecrosis (left). (**f**) Diffuse strong cytoplasmic staining of neoplastic cells with pan-CKs AE1-AE3, DAB Chromogen. (**g**) Diffuse, intermediate-to-high intensity staining of neoplastic cells with CK7, DAB Chromogen. (**h**) Multifocal, low-to-moderate staining of neoplastic cells with CK20, DAB Chromogen.

Cytological smears from the pericardial effusion were composed of numerous cohesive clusters of cuboidal cells (Figure 4a). Occasional acinar structures with a central amorphous pink–violet material were observed. Neoplastic cells exhibited an intermediate to high nucleus-to-cytoplasm ratio, scant and occasionally vacuolated cytoplasm, round paracentral nuclei with unevenly granular chromatin, variably visible nucleoli, and moderate anisokaryosis. These findings were consistent with an epithelial neoplasm. A mixed inflammatory infiltrate consisting mainly of neutrophils and macrophages was also present along with yellowish crystals (consistent with hematoidin and bilirubin).

Histologically, the liver showed an infiltrative, non-capsulated neoplastic epithelial neoplasm (Figure 4b) composed of tubular and acinar structures embedded in a variable amount of desmoplastic stroma. Neoplastic cells were cuboidal to low columnar, with scant eosinophilic cytoplasm and a large, round nucleus, with vesicular chromatin and variably prominent central nucleoli. The mitotic range was 0–5 per high power field (0.237 mm^2^). The neoplastic tubules and acini contained proteinaceous material, necrotic debris, and sporadic yellowish material, consistent with biliary pigment (Figure 4b, inset). Extensive areas of necrosis and hemorrhage were noted. Similar neoplastic lesions were also observed in the diaphragm (on both the pleural and peritoneal surfaces) (Figure 3c), mesentery, spermatic cord, kidney, spleen, pericardium, and kidney. Neoplastic lymphovascular invasion was observed (Figure 4d), most frequently in the diaphragmatic metastatic nodules, and confirmed with von Willebrand factor (vWF) immunohistochemistry (IHC) for endothelial cells (Figure 4d inset).

The neoplastic cells in hepatic sections displayed diffuse strong cytoplasmic staining with pan-CKs (Figure 4f), diffuse CK7 staining of intermediate-to-high intensity (Figure 4g), and multifocal, low-to-moderate CK20 staining (Figure 4h). In the metastatic nodules, the pan-CKs were diffusely positive, while CK7 staining was less frequent and less intense, with a greater decrease in CK20 expression.

The histologic and immunohistochemical findings were consistent with an intrahepatic metastatic CCA.

### 2.4. Microbiological Findings

A microbiological examination for aerobic and anaerobic microorganisms was performed on specimens and swabs collected during the post-mortem examination. Tissue aspirates were collected from the liver and intestinal contents with sterile swabs (Copan Italia S.p.A., Brescia, Italy). Samples were then diluted in 1 mL of a nutrient broth (HIB, Heart Infusion Broth, Conda, Madrid, Spain), and 10 and 100 μL of bacterial suspensions were then inoculated into solid media and broths, respectively, as described below. The evaluation of aerobic microorganisms was conducted using a nutrient medium (BA, Blood Agar Base n°2, Biolife, Milan, Italy) with 5% defibrinated sheep blood (Allevamento Blood, Teramo, Italy), a nutrient broth (HIB), and selective *Enterobacteriaceae* media (McConkey agar, Oxoid, Basingstoke, UK). Cultures were inoculated and incubated at 37 ± 1 °C in aerobic conditions. An assessment of anaerobic microorganisms was performed using a nutrient medium (BA), selective media for *C. perfringens* (TSC Agar Base, Biolife, Milan, Italy), and a fluid Thioglycollate medium (THG, Liofilchem, Roseto degli Abruzzi, Teramo, Italy). The cultures were inoculated and incubated at 37 ± 1 °C under anaerobic conditions. *E. coli* was detected at low levels in the liver samples. From the intestinal swabs, coagulase-negative Staphylococci, *E. coli,* and *C. perfringens* were isolated.

A section of intestine (approximately 5 mm^3^) was homogenized in 0.8 mL of Phosphate-Buffered Saline (PBS) supplemented with antibiotics (PBS-A: 10,000 IU/mL penicillin G, 10 mg/mL streptomycin, 5000 IU/mL nystatin, and 0.25 mg/mL gentamicin sulfate) using a Tissue Lyser (QIAGEN, Hilden, Germany) at 30 Hz for 3 min. One hundred microliters (100 µL) of the intestinal homogenate was used for viral DNA/RNA extraction, which was performed with the KingFisher™ Flex Purification System (Life Technologies, Carlsbad, CA, USA) and the ID Gene^®^ Mag Universal Extraction Kit (IDvet, Grabels, France), following the manufacturer’s instructions. The extracted DNA was tested for feline parvovirus (FPV) using a Real-Time PCR, targeting a region of the VP2 gene [9] with the QuantiFast Pathogen PCR + IC Kit (QIAGEN, Hilden, Germany). Meanwhile, the extracted RNA was tested for feline coronavirus (FCoV) using a Real-Time RT-PCR with the VetMAX™ FIP Dual IPC Kit (Thermo Fisher Scientific, Waltham, MA, USA). All molecular assays were performed on the CFX 96 Deep Well Real-Time PCR system (BioRad Laboratories Inc., Hercules, CA, USA). The intestinal sample tested negative for FPV and positive for FCoV in the Real-Time PCR and Real-Time RT-PCR assays [10].

## 3. Discussion

Captive populations of large felids often exhibit high stability due to dedicated care and long-term monitoring [1,11]. These animals are susceptible to several disorders caused by environmental influences, infectious agents, and genetic factors. These diseases can ultimately lead to the decline of these populations and the effectiveness of conservation strategies. Furthermore, it is important to assess the risk of zoonotic diseases affecting large cats in captivity, as they have the potential for a significant impact on human health both as environmental sentinels and susceptible hosts for infectious or zoonotic diseases [12,13,14,15,16,17,18,19,20,21].

Neoplastic diseases represent an important cause of morbidity and mortality in captive felids [1,5,22,23,24,25,26]. The increased longevity of wild captive animals is likely to contribute to the increased incidence of cancer in captive felids compared to their free-ranging counterparts [5,27]. In addition, the increased exposure to environmental pollutants, infectious agents, and genetic predisposition may promote the development of neoplastic disease [1,28,29].

Intrahepatic CCAs are malignant tumors that arise from the biliary duct epithelium and present either as a single mass or as coalescing, nodular proliferations on the surface of the liver and throughout the parenchyma [30]. Although primary hepatobiliary tumors are rare in domestic animals, CCAs seem to be the most common non-hematopoietic hepatic tumors in domestic cats [31]. In cats, CAAs originating from intrahepatic bile ducts are far more common than those arising from extrahepatic bile ducts or the gallbladder [30,32].

Cholangiocarcinoma typically occurs in domestic cats over 10 years of age and no consistent breed or sex predisposition has been reported [30]. However, some authors have suggested that there may be a higher prevalence in male cats [28,33].

Given the paucity of available reports, intrahepatic or extrahepatic CAA appears to be an infrequently documented condition in captive felids and is often associated with widespread metastases, although it may be underdiagnosed [3,6,34], and is often associated with widespread metastases. CCAs exhibit a highly invasive growth pattern and frequently metastasize in all species, with a reported metastasis rate of 78% in cats, according to one survey [30,35]. In captive pumas, digestive neoplasms, including hepatic tumors, are the most commonly reported after endocrine and neuroendocrine neoplasms [4]. Although rare cases of benign hepatic cholangioma have been documented, to the best of the author’s knowledge, only a single case report of intrahepatic CCA in a captive puma has been reported thus far [4,6]. Similar to the present report, this case presented with a primary tumor characterized by both massive and multinodular growth, and multiorgan metastases to the lung, stomach, kidney, heart, and diaphragm [6].

In our case report, the histological appearance of the tumor, with tubular and acinar structures, allowed for the exclusion of hepatocellular carcinoma as a differential diagnosis, supported by the immunoreactivity to pan-CKs, and CK7 [36]. While canine CCAs have demonstrated a CK7+/CK20- immunophenotype, CK20 expression has been reported in the feline species, similar to our case [37].

In this case report, an elderly captive puma presented with mild gastrointestinal signs (vomiting and diarrhea), abdominal distension, and subtle respiratory changes. In the initial clinical assessment, the signs appeared relatively mild and non-specific. However, after sedation, palpation and a diagnostic evaluation revealed a far more severe clinical picture with chronic multiorgan involvement. Imaging studies revealed the presence of multiple cavitary effusions—including abdominal, pleural, and pericardial fluid—along with significant cardiopulmonary compromise. Other cardiac abnormalities included atrioventricular valve insufficiency, likely due to degenerative valve disease, and atrial fibrillation.

According to the recent literature [13,38,39,40], the hematology and biochemical findings indicate a mild increase in the total RBC count, as well as the hemoglobin and hematocrit values, which, contextualized within the clinical picture, may be associated with a mild to moderate state of dehydration [41]. Cell morphology reveals alterations indicative of an inflammatory or infectious state (anisocytosis, echinocytosis, neutrophil toxicity, Döhle bodies, and monocytosis) [42]. The biochemical parameters do not show any particular alterations. The intestine tested positive for FCoV and negative for FPV. Feline coronavirus is a ubiquitous RNA virus present in cats and non-domestic felid populations worldwide. FCoV is primarily an enteric virus, and infection is often asymptomatic or presents as enteritis, although outbreaks of feline infectious peritonitis (FIP) have been reported in captive felids [43,44,45]. In this report, the presence of FCoV, in the absence of FIP-related lesions, suggests an infection confined to the intestine, which may or may not have contributed to the gastrointestinal signs.

The etiology of bile duct tumors remains unclear, although several risk factors have been proposed to play a role in the development of CAAs in domestic animals and humans, including chronic inflammation of the biliary tract, cholestasis, fluke infestation, or chemical agents [46]. In humans, infection with the liver flukes *Opisthorchis viverrini* and *Clonorchis sinensis* has been recognized a risk factor for CCA in Southeast Asia [47,48]. Some studies in cats have suggested a similar association with *Clonorchis sinensis* infestation, but the limited data make it difficult to draw conclusions about the role of parasitism in the development of CCA in this species [30,49]. Ascarid, such as those caused by *Toxascaris leonina*, are not currently recognized as risk factors for hepatic neoplasia in felids. Carcinogenic chemicals, such as furans and nitrosamines, have been shown to induce CCA in laboratory rodents and dogs, respectively [50,51]. Chronic inflammation, regardless of cause, may stimulate increased biliary epithelial cell replication, which could facilitate tumor development over time [30]. In a survey of hepatic lesions in captive felids, non-neoplastic biliary tract alterations including biliary hyperplasia, cysts, and cholestasis were present in 35% of pumas, and portal hepatitis was present in 24% of cases [25,30]. However, no evidence of predisposing factors was identified in the present case.

## 4. Conclusions

To the best of our knowledge, this is the first reported case of CCA in a captive puma, documented through a comprehensive diagnostic investigation (ante- and post-mortem). The effective management of captive wild felids requires tailored diagnostic protocols to ascertain a diagnosis, both in vivo and post-mortem. A multidisciplinary team comprising veterinary clinicians, anesthetists, radiologists, infectious disease specialists, and pathologists is essential. Furthermore, although the expertise of individual professionals is highly regarded, a knowledge gap emerges when considering the specific characteristics and management of chronic pathologies in these animals.

## Figures and Tables

**Table 1:** Hematological analyses were performed with the Sysmex XN1000-V analyzer (Sysmex Europe GmbH, Norderstedt, Germany). Biochemistry analyses were performed on serum through the Cobas c501 clinical chemistry analyzer with the related kit (Roche Diagnostics GmbH, Mannheim, Germany).

Hematology	Value
RBCs	9.96 M/μL
Hgb	14.8 g/dL
Hct	41.8%
MCV	42.0 fL
MCH	14.9 pg
MCHC	35.4 g/dL
RDW	21.2%
RBCs Morphology	Anisocytosis, Echinocytosis
WBCs	14.94 (10^3^/μL)
NEUT	13.13 (10^3^/μL)
LYMPH	0.72 (10^3^/μL)
MONO	1.02 (10^3^/μL)
EO	0.06 (10^3^/μL)
BASO	0.01 (10^3^/μL)
WBCs Morphology	Neutrophil Toxicity, Döhle bodies
PLT	329 (10^3^/μL)
MPV	9.7 fL
**Serum Biochemistry**	**Value**
Total Proteins	71 g/L
Albumin	34 g/L
Globulin	37 g/L
Urea Nitrogen	25.2 mmol/L
Creatinine	284 μmol/L
Glucose	3.1 mmol/L
Cholesterol	4.25 mmol/L
Triglycerides	0.50 mmol/L
Total Bilirubin	4.84 μmol/L
Direct Bilirubin	2.55 μmol/L
Unconjugated Bilirubin	2.29 μmol/L
AST	50 U/L
ALT	34 U/L
ALP	19 U/L
GGT	8 U/L
Cholinesterase	4385 U/L
CK	229 U/L
Calcium	2.58 mmol/L
Phosphorus	1.65 mmol/L
Magnesium	1.27 mmol/L
Sodium	156 mmol/L
Potassium	4.53 mmol/L
Chlorine	115 mmol/L
Iron	55 μg/dL

RBCs (Red Blood Cells), Hgb (Hemoglobin), Hct (Hematocrit), MCV (Mean Corpuscular Volume), MCH (Mean Corpuscular Hemoglobin), MCHC (Mean Corpuscular Hemoglobin Concentration), RDW (Red Cell Distribution Width), WBCs (White Blood Cells), NEUT (Neutrophils), LYMPH (Lymphocytes), MONO (Monocytes), EO (Eosinophils), BASO (Basophils), PLT (Platelets), MPV (Mean Platelet Volume), AST (Serum Glutamic Oxaloacetic Transaminase), ALT (Serum Glutamic Pyruvic Transaminase), ALP (Alkaline Phosphatase), GGT (Gamma Glutamil Transferase), CK (Creatine Kinase).

**Table 2:** Antibody panels and immunohistochemistry protocols applied to the intrahepatic metastatic cholangiocarcinoma of a captive puma.

Antibody	Antigen Retrieval	Antibody Incubation	Detection System
Pan cytokeratin	CC1 ^a,b^; 95 °C; 32 min	1:50; 24 min; RT	Discovery OmniMap anti Mouse HRP ^b^ 16 min; Discovery Chromomap DAB ^b^
CK7	CC1; 95 °C; 64 min	1:20; 1 h; RT	Discovery Anti Mouse HQ ^b^ 36 °C 8 min; Discovery anti HQ HRP ^b^ 8 min; Discovery Chromomap DAB
CK20	CC1; 95 °C; 40 min	1:20; 1 h; 37 °C	Discovery Anti Mouse HQ 36 °C 8 min; Discovery anti HQ HRP 8 min; Discovery Chromomap DAB
von Willebrand factor (vWF)	Protease 2; 36 °C; 8 min	1:200; 32 min; RT	Discovery OmniMap anti Rabbit HRPb 16 min; Discovery Chromomap DAB ^b^

DAB, 3′-3′-Diaminobenzidine; HRP, Horseradish Peroxidase; RT, room temperature. ^a^ CC1: cell conditioning solution 1 (pH 8.4). ^b^ Roche, Ventana Medical Systems (Tucson, AZ, USA).

## Data Availability

All the data are available in the present manuscript.
